# Breakthrough infections and waning immune responses with ChAdOx1 nCoV‐19 or mRNA vaccine in healthcare workers

**DOI:** 10.1002/ctm2.804

**Published:** 2022-04-22

**Authors:** So Yun Lim, Jiwon Jung, Ji Yeun Kim, Soonju Park, Ji‐Soo Kwon, So Yeon Park, Sun‐Kyung Kim, Young‐Ju Lim, Eun Ok Kim, Seongman Bae, Min Jae Kim, Yong Pil Chong, Sang‐Oh Lee, Sang‐Ho Choi, Yang Soo Kim, Nakyung Lee, Kideok Kim, David Shum, Youngmee Jee, Sung‐Han Kim

**Affiliations:** ^1^ Department of Infectious Diseases Asan Medical Center University of Ulsan College of Medicine Seoul Republic of Korea; ^2^ Office for Infection Control Asan Medical Center University of Ulsan College of Medicine Seoul South Korea; ^3^ Institut Pasteur Korea Seongnam‐si Republic of Korea

Dear Editor,

Breakthrough infection of SARS‐CoV‐2 has been increased according to the emergence of the delta variant and vaccine‐induced waning immunity.^1^ In addition, epidemiologic studies have reported that people with previous SARS‐CoV‐2 infection show decreasing antibody levels against SARS‐CoV‐2^2^ and can be reinfected with the virus later on.^3^ Here, we assessed the waning vaccine effectiveness by comparing the breakthrough infection rate between healthcare workers (HCWs) vaccinated with two doses of ChAdOx1 nCoV‐19 (ChAdOx1) or mRNA vaccine. We also compared immune responses against ancestral SARS‐CoV‐2 and the delta variant between individuals with natural infection and HCWs who received the ChAdOx1 or BNT162b2 vaccine.

This study was performed at Asan Medical Center with 15,034 HCWs, Seoul, South Korea. First, we retrospectively compared the breakthrough infection rate in HCWs who were vaccinated with two doses of ChAdOx1 or mRNA vaccine. We evaluated the infection rates stratified by 30‐ or 60‐day interval and by vaccine type in each period. Second, we conducted a 6‐month longitudinal prospective study in HCWs who either received two doses of

ChAdOx1 or BNT162b2 vaccines and individuals with previous SARS‐CoV‐2 infection. The detailed methods for enrolment, evaluation of breakthrough infection rates and measurement of immune responses are described in Supporting Information.

As of December 15, there were 14,427 fully vaccinated HCWs (ChAdOx1, n = 9717; mRNA‐1273, n = 1441; BNT162b2, n = 1297; other n = 1972). Most of the HCWs (82%, 7966/9717) vaccinated with the ChAdOx1 vaccine received their first dose in March, and most of the HCWs vaccinated with the mRNA vaccine received their first dose between June and September (84%, 2305/2738, Figure 1A). Of the 12,455 HCWs who were fully vaccinated with ChAdOx1 or mRNA vaccine, 105 (0.8%) had breakthrough infections. All breakthrough infection cases were asymptomatic or mild illness. Of these, 67 (64%) were females (median age was 36; interquartile range [IQR 28‐46]), and 90 (86%) were vaccinated with ChAdOx1 nCoV‐19. The number of infections has increased since October (Figure 1B). The infection rate increased as time passed after the first vaccination (*P* for trend < .001 in the ChAdOx1 group and < .007 in the mRNA vaccine group, respectively, Figure 1C, Table S1). There was no difference in the infection rate between the ChAdOx1 vaccine and the mRNA vaccine in each period (.06% vs. .08% in 91‐120 days, *P* = .69;.01% vs..09% in 121‐150 days, *P *= .10; .08% vs. .11% in 151‐180 days, *P* = .66; .09% vs..21% in 181‐210 days, *P *= .26; .19% vs. .54% in 211‐240 days; *P* = .17; .63% vs. .55%; *P *> .99).

**FIGURE 1:**
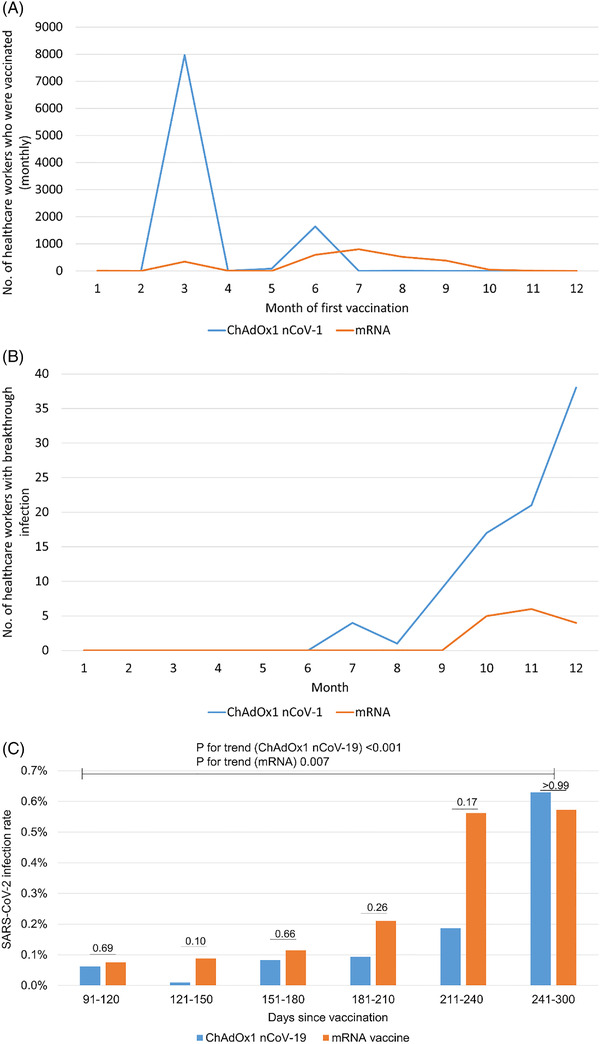
(A) Monthly number of healthcare workers (HCWs) who were vaccinated. (B) Monthly number of HCWs with breakthrough infection. (C) The infection rate of HCWs fully vaccinated with ChAdOx1 nCoV‐19 and mRNA vaccine stratified by days since first vaccination

Additionally, a total of 117 HCWs who received the ChAdOx1 vaccine (n = 82 [70%]) or BNT162b2 vaccine (n = 35 [30%]) and 28 participants with natural SARS‐CoV‐2 infection were enrolled in this study. Of these 117 HCWs, there were no breakthrough infections during the study period. The baseline characteristics of the natural infection group and the two vaccination groups are shown in Table 1. In the natural infection group, the median titer of S1‐specific IgG peaked at 1 month after infection and significantly decreased by 6 months (1252‐106.8 IU/ml, Figure 2A). At peak immunity, the S1‐specific antibody titer was significantly higher in the BNT162b2 group than in the natural infection group; however, the titres were comparable between the two groups at 6 months. The ChAdOx1 group showed a significantly lower S1‐specific antibody response than the natural infection group or the BNT162b2 group both at peak immunity and at 6 months. The detailed kinetics of the S1‐specific antibody titer over 6 months in the three groups are shown in Figure 3A and Figure S1. The neutralising antibody titres to the ancestral strain were more durable over 6 months in the natural infection group (from 2406 at peak to 1870 at 6 months; *P *= .21) than in the vaccination groups, with a significant decrease in antibody titres (both *P* < .001, Figure 2B). The detailed kinetics of neutralising antibody titres to the ancestral strain over 6 months in the three groups are shown in Figure 3B. The neutralising antibody titres to the delta variant at 6 months were significantly higher in the natural infection group (391.1) than in the BNT162b2 (166.5, *P *= .003) and ChAdOX1 (110.6, *P *< .001) groups, while the titres in the two vaccination groups were comparable (*P *= .17; Figure 2C). A comparison of antibody responses according to the severity of SARS‐CoV‐2 infection is shown in the Extended Results section of Supporting Information. Interferon‐γ‐producing T‐cell responses showed a decreasing trend over 6 months in all three groups. At the peak immune response, the BNT162b2 group showed the highest T‐cell response (155 SFC/500,000 PBMCs) compared with the natural infection group (50.50 SFC/500,000 PBMCs; *P *= .02) or the ChAdOx1 group (84.50 SFC/500,000 PBMCs; *P* = .26), while the T‐cell responses in the natural infection group and the BNT162b2 group were comparable at 6 months (*P *= .63; Figure 2D, Figure S2).

**TABLE 1:** Baseline characteristics of the vaccination group and study participants with natural SARS‐CoV‐2 infection according to disease severity

Vaccination group	ChAdOx1 (*n* = 82)	BNT162b2 (*n* = 35)	*P* value
Age at vaccination, years, median (range)	37 (21‐64)	32 (24‐53)	.003
Age range			.04
20s	20 (24)	17 (49)	
30s	32 (39)	14 (40)	
40s	20 (24)	3 (8.6)	
50s	8 (9.8)	1 (2.9)	
60s	2 (2.4)	0 (0)	
Sex			.24
Female	64 (78)	23 (66)	
Male	18 (22)	12 (34)	

Natural infection	Mild or moderate (*n* = 11^a^)	Severe or critical (*n* = 17)	*P* value
Age, years, median (range)	35 (33‐70)	70 (35‐89)	.036
Sex			.002
Female	11 (100)	7 (41)	
Male	0 (0)	10 (59)	
Mortality	0 (0)	3 (18)	.26
Hospital day, median (range)	9 (5‐24)	14 (9‐140)	.18

*Note*: Data represent *n* (%) unless indicated otherwise.

^a^ Included one asymptomatic patient.

FIGURE 2Humoral and cell‐mediated immune responses over 6 months after SARS‐CoV‐2 natural infection and COVID‐19 vaccination. Solid lines indicate the median values. (A) S1‐specific IgG antibody. (B) Neutralising antibody against ancestral strain. (C) Neutralising antibody against the delta strain. (D) S1‐specific T‐cell response

**Figure:**
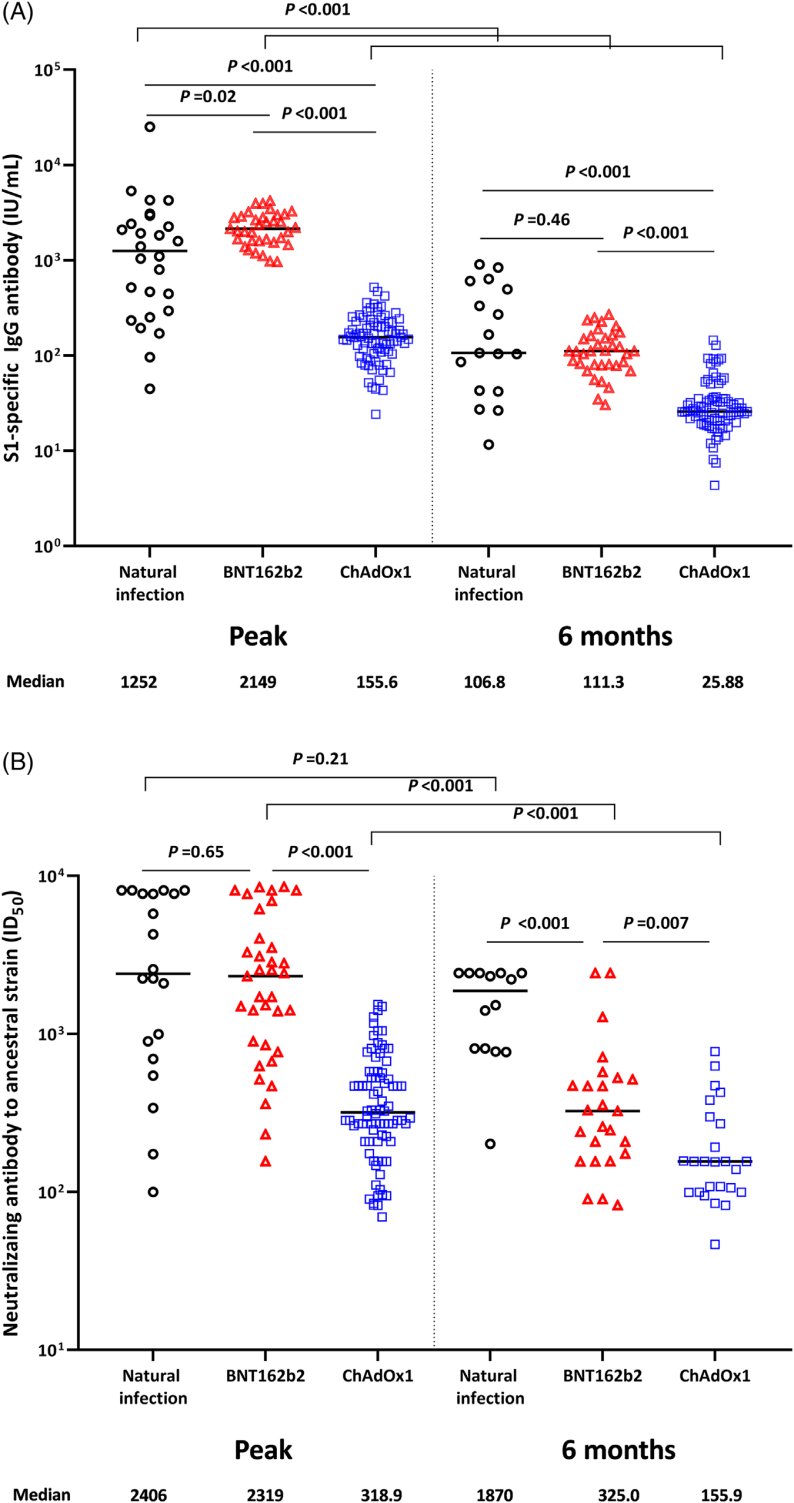

**Figure:**
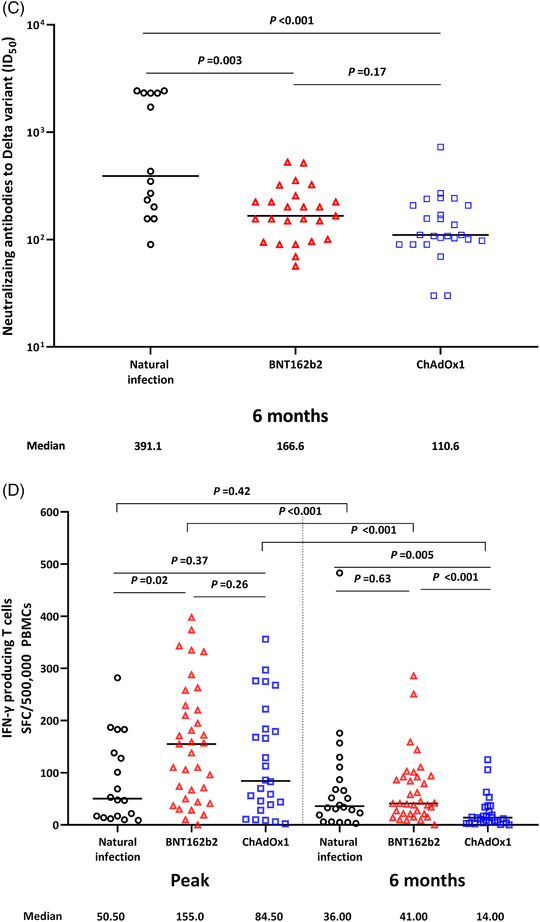

**FIGURE 3:**
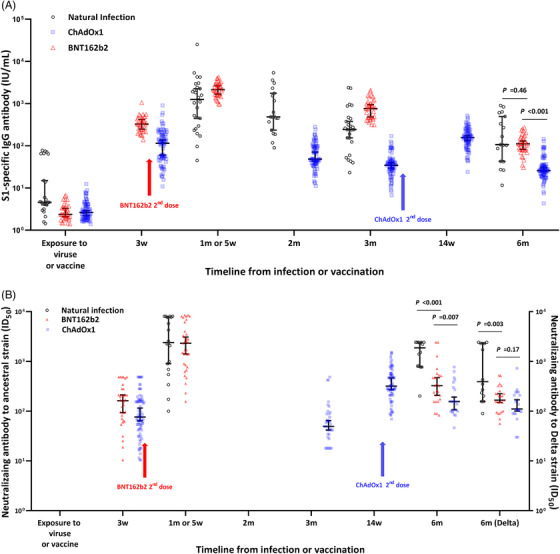
Kinetics of the antibody response induced by SARS‐CoV‐2 infection and COVID‐19 vaccination. Solid lines indicate the median values, and the error bars indicate the 95% confidential intervals. (A) Kinetics of S1‐specific IgG antibody. (B) Kinetics of neutralising antibody

Data regarding the duration of the protective effect after ChAdOx1 are limited. In a population‐based cohort study from Brazil and Scotland, hospital admissions and death increased 3 months after the second dose of ChAdOx1,^4^ suggesting the waning vaccine effectiveness induced by ChAdOx1 vaccination. Moreover, there are a few comparative data for waning immunity between the two vaccine platforms. In this study, we found that the breakthrough infection rate increased as time passed after vaccination, but there was no significant difference in the breakthrough infection rate between HCWs who received ChAdOx1 and those who received mRNA vaccines. Our findings were further consolidated by the results that neutralising antibody levels against the delta variant after BNT162b2 vaccination declined rapidly and were similar at 6 months between the BNT162b2 group and the ChAdOx1 group. However, the antibody titres at 6 months, especially those against the delta variant, were significantly higher in the natural infection group than in either of the COVID‐19 vaccination groups, which implies the longevity and robustness of the immune response after natural infection compared with COVID‐19 vaccination. Our findings are consistent with recent epidemiological studies that reported long‐term protective immunity after SARS‐CoV‐2 infection, with a more than 80% reduction in the risk of reinfection over 7 months following primary SARS‐CoV‐2 infection.^3^, ^5^, ^6^

The relatively small sample size of immune response measurements and the different study participants between the two cohorts in our study may limit further interpretation of our results. Additionally, SARS‐CoV‐2 variant identification was not performed in our study. However, most of the breakthrough infections occurred after August 2021, when the delta variant was dominant in South Korea. Therefore, variants other than the delta variant did not significantly affect our study. In conclusion, we found that the rate of breakthrough infection in ChAdOx1‐vaccinated HCWs was similar to that in mRNA‐vaccinated HCWs in the delta variant‐dominant area despite some different immunogenicity. In addition, neutralising antibody levels against the delta variant after COVID‐19 vaccination appear to decline more rapidly than those after natural SAR‐CoV‐2 infection.

## CONFLICTS OF INTEREST

There are no conflicts of interest for any of the authors.

## Supporting information

Supporting information

Supporting information

Supporting information

Supporting information

Supporting information

Supporting information

Supporting information

Supporting information

Supporting information

Supporting information

Supporting information

Supporting information
