# Supplementary material for: Breakthrough infections and waning immune responses with ChAdOx1 nCoV‐19 or mRNA vaccine in healthcare workers
Source: Clin Transl Med. 2022 Apr 22;12(4):e804. doi: 10.1002/ctm2.804 (PMC9029012; doi:10.1002/ctm2.804)
Supplement: Supplementary file 1 — Supporting information [file CTM2-12-e804-s006.docx]

**Supplemental materials**

**Detailed methods**

***Evaluation of breakthrough infection rates in total HCW’s cohort***

This study was performed in Asan Medical Center, a tertiary care hospital with 15,034 HCWs, Seoul, South Korea. From March 5, 2021, HCWs began receiving vaccinations at the COVID-19 vaccine center in our hospital. Frontline HCWs including doctors and nurses directly treating COVID-19 patients were vaccinated with BNT162b2, and other licensed HCWs (doctor, nurse, nursing assistant, pharmacist, and other paramedics) were vaccinated with ChAdOx1 first. Afterwards, other hospital staff were vaccinated with mRNA vaccine depending on the vaccine availability in South Korea. The intervals between the two doses of ChAdOx1, BNT162b2, and mRNA-1273 vaccines were 12 weeks, 3 weeks, and 4 weeks, respectively. We retrospectively reviewed the data of vaccine type, the date of vaccination, and HCWs who were positive for SARS-CoV-2 by RT-PCR. Full vaccinated HCWs with ChAdOx1 or mRNA vaccine were included in this study. Full vaccination was defined as those who were vaccinated ≥ 14 days before the primary series of COVID-19 vaccine as of December 15, 2021 or those completed the primary series of COVID-19 vaccine ≥ 14 days before a receipt of a positive SARS-CoV-2 test result. We divided the period after first vaccination date (91-120, 121-150, 151-180, 181-210, 211-240, and 241-300 days after first vaccination date) and breakthrough infection rates were stratified by the period. In addition, the infection rates were compared by vaccine type in each period. Breakthrough infection was defined as SARS-CoV-2 infection confirmed by SARS-CoV-2 PCR from a respiratory specimen from a person 2 weeks or more after 2-dose of ChAdOx1 or mRNA vaccine^1^.

***Evaluation of immune responses in a prospective cohort study***

We enrolled healthcare workers (HCWs) between March 5^th^ and March 25^th^, 2021 at a tertiary care hospital in Seoul, South Korea, who received the ChAdOx1 or BNT162b2 vaccines without a history of SARS-CoV-2 infection before vaccination as confirmed by negative S1-specific antibody results in ELISA. Blood sampling before vaccination was used as the baseline sample and other samples were acquired during scheduled follow-up. Recruitment for the study was done by voluntary participation through a posting on the Asan Medical Center intranet.

We also enrolled individuals with previous SARS-CoV-2 natural infection between March 2020 and February 2021 when COVID-19 vaccines were unavailable in South Korea. Participants with natural infection were excluded from analysis if they had received any kind of COVID-19 vaccination after study enrollment and were classified according to the severity of illness according to the NIH classification as follows: asymptomatic or presymptomatic infection=1, mild illness=2, moderate illness=3, severe illness=4, critical illness=5.

***Measurement of immune responses***

SARS-CoV-2 S1-specific IgG antibody titers were measured using in-house developed enzyme-linked immunosorbent assay (ELISA) qualified with reference pooled sera from International Vaccine Institute (Seoul, South Korea) and the S1 specific IgG antibody titers have been re-presented as International Units per milliliter (IU/ml). To determine cut-off values for the ELISA, the mean and standard deviation (SD) of negative control plasma were measured, and cut-off values were defined as mean IU plus three-fold the SD value; the cut-off value was 10 IU/ml for IgG, according the previous studies^2-4^.

Plasma levels of live-virus neutralizing antibodies were measured using a microneutralization assay. Briefly, 100 tissue culture infective dose 50 (100 TCID50) of SARS-CoV-2 ancestral strain (NCCP 43326 Human corona virus, BetaCoV/Korea/KCDC03/2020) or delta variants (NCCP 43405 Human corona virus, BetaCoV/Korea/KCDC03/2020), approved by the Centers for Diseas Control and Prevention, Korea, was mixed with diluted plasma specimen in equal volume, incubated at 37°C for 30 minutes, and added to Vero cells. After 96 hours, the cytopathic effect of SARS-CoV-2 on the infected cells was measured. Neutralization antibody titer was presented as the reciprocal of the highest test plasma dilution factors at which 50% neutralization is attained (ID50). The MN assay was performed in a Bio Safety Level (BSL)-3 laboratory in Institut Pasteur Korea (Seongnam, Republic of Korea). The correlation between the 50% neutralizing antibody titer and S1-specific IgG antibody titer was presented in Appendix Figure 1.

An IFN-gamma enzyme-linked immunospot (ELISPOT) assay was performed to measure the SARS-CoV-2-specific T cell response from PBMCs isolated from blood samples of the participants. T cells were stimulated with overlapping peptides of the SARS-CoV-2 spike protein (Miltenyi Biotec, Bergisch Gladbach, Germany) and numbers of spot-forming cells (SFC) per 5.0×105 PBMCs were counted with an automated ELISPOT reader (AID iSPOT, Autoimmun Diagnostika GmbH, Strassberg, Germany). We performed ELISPOT assay with pool of overlapping peptides, mainly consisting of 15-mer sequences with 11 amino acids overlap, covering protein coding sequence of the spike glycoprotein of SARS-CoV-2. The final concentration of pooled peptides for stimulation was 1 μg/mL per peptide.

***Statistical analysis***

We used the Cochran-Armitage test for trend for evaluating the waning immunity after vaccination. We used the Chi-squared test or Fisher’s exact test for the analysis of categorical variables. Student’s t-test or Mann Whitney U test was used for the continuous variables according to the normality of the data. Two-tailed P values < .05 were considered statistically significant. R version 4.1.1 (R Project for Statistical Computing, Vienna, Austria) and Graphpad Prism version 8.0 (GraphPad Software, San Diego, CA, USA) were used for the analysis and graph plotting of the results.

***Patient Consent Statement***

The patient’s written consent was obtained. The study was conducted according to the guidelines of the Declaration of Helsinki and approved by the Institutional Review Board of Asan Medical Center (IRB No. 2020-0297, IRB No. 2021-0170, and 2021-0024).

**Extended Results**

***Comparison of antibody responses according to the severity of SARS-CoV-2 infection***

When compared with participants who were asymptomatic or had mild-to-moderate illness, those who had experienced severe-to-critical illness showed higher antibody responses at 6 months after infection in terms of S1-specific IgG antibody (asymptomatic-to-moderate, 56.69 IU/mL; severe-to-critical, 496.8 IU/mL; *P* = 0.005, Appendix Figure 2A), and neutralizing antibody to the delta variant (asymptomatic-to-moderate, 156.4; severe-to-critical, 1716; *P* = 0.02, Appendix Figure 2C) but not to the ancestral strain (P = 0.27, Appendix Figure 2B).

Reference

1. CDC, COVID-19 Vaccine Breakthrough Case Investigation Team. COVID-19 Vaccine Breakthrough Infections Reported to CDC - United States, January 1-April 30, 2021. *MMWR Morb Mortal Wkly Rep*. May 28 2021;70(21):792-793. doi:10.15585/mmwr.mm7021e3

2. Classen DC, Morningstar JM, Shanley JD. Detection of antibody to murine cytomegalovirus by enzyme-linked immunosorbent and indirect immunofluorescence assays. *J Clin Microbiol*. Apr 1987;25(4):600-4. doi:10.1128/jcm.25.4.600-604.1987

3. Lardeux F, Torrico G, Aliaga C. Calculation of the ELISA's cut-off based on the change-point analysis method for detection of Trypanosoma cruzi infection in Bolivian dogs in the absence of controls. *Mem Inst Oswaldo Cruz*. Jul 4 2016;111(8):501-4. doi:10.1590/0074-02760160119

4. Kim JY, Lim SY, Park S, Kwon JS, Bae S, Park JY et al. Immune responses to the ChAdOx1 nCoV-19 and BNT162b2 vaccines and to natural COVID-19 infections over a three-month period. *J Infect Dis*. 2021 Nov 25:jiab579. doi: 10.1093/infdis/jiab579
