# Supplementary figures and images for: Breakthrough infections and waning immune responses with ChAdOx1 nCoV‐19 or mRNA vaccine in healthcare workers
Source: Clin Transl Med. 2022 Apr 22;12(4):e804. doi: 10.1002/ctm2.804 (PMC9029012; doi:10.1002/ctm2.804)

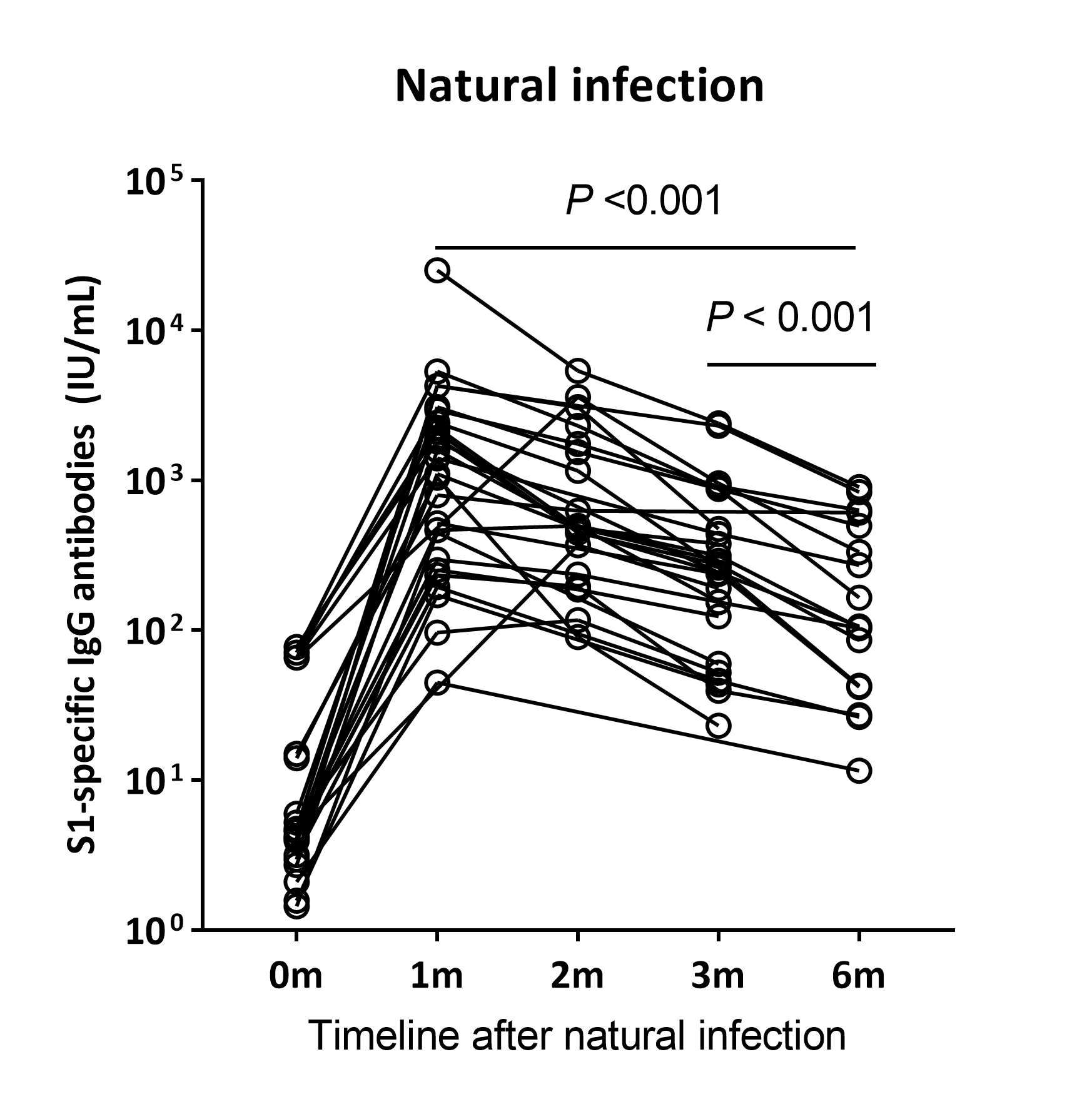

Supplement: Supplementary file 2 — Supporting information [file CTM2-12-e804-s002.tif]

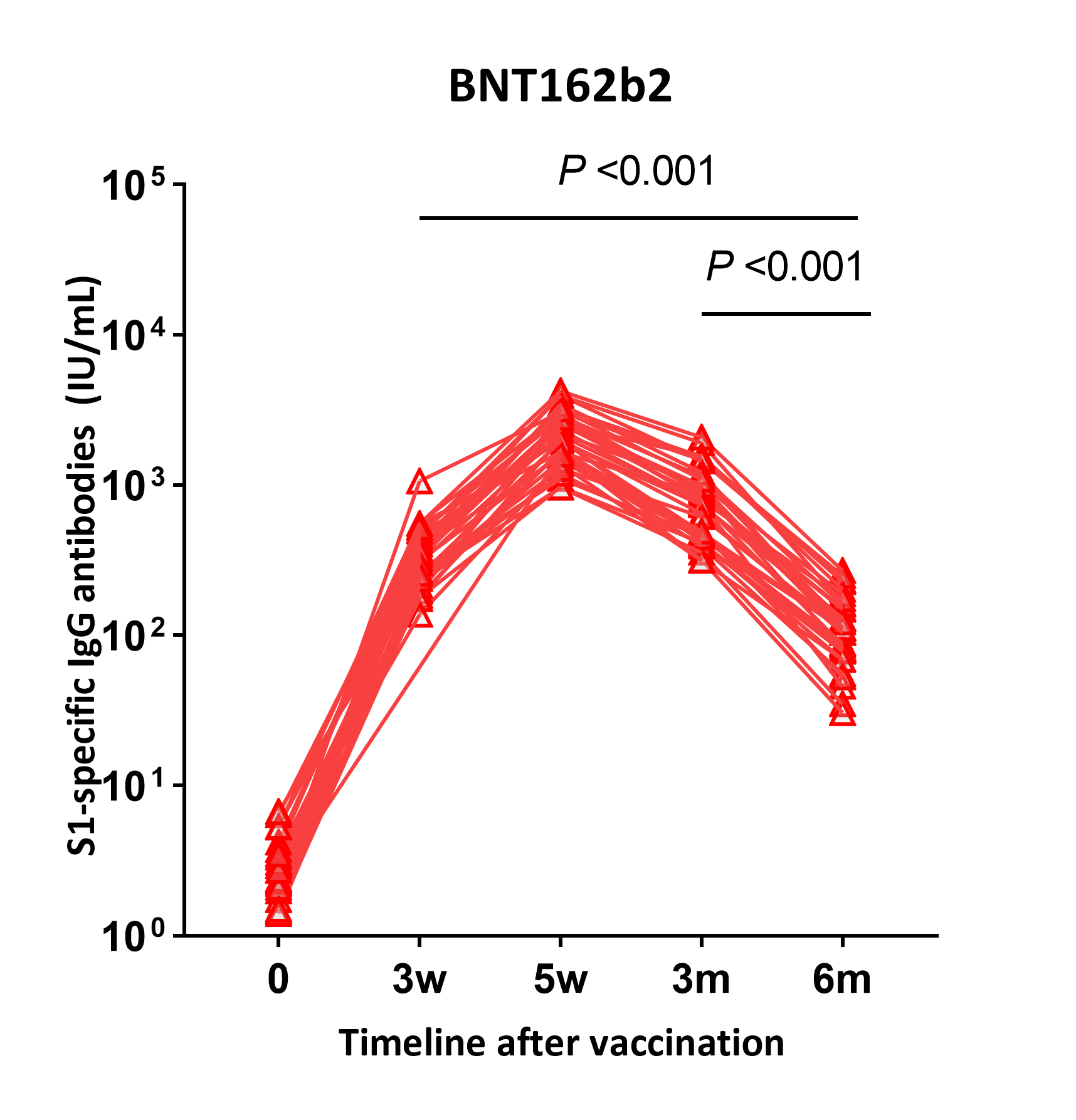

Supplement: Supplementary file 3 — Supporting information [file CTM2-12-e804-s012.tif]

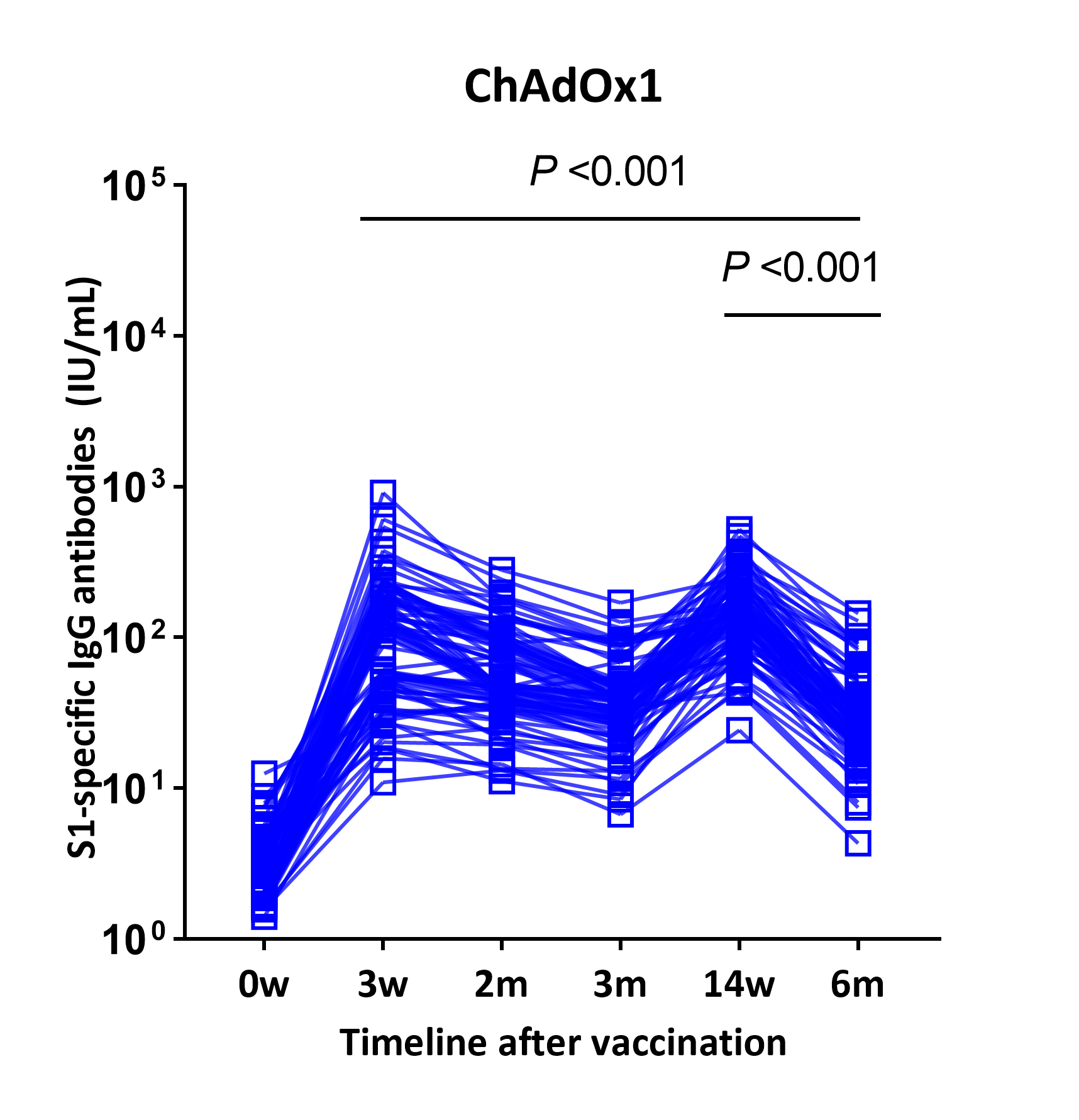

Supplement: Supplementary file 4 — Supporting information [file CTM2-12-e804-s009.tif]

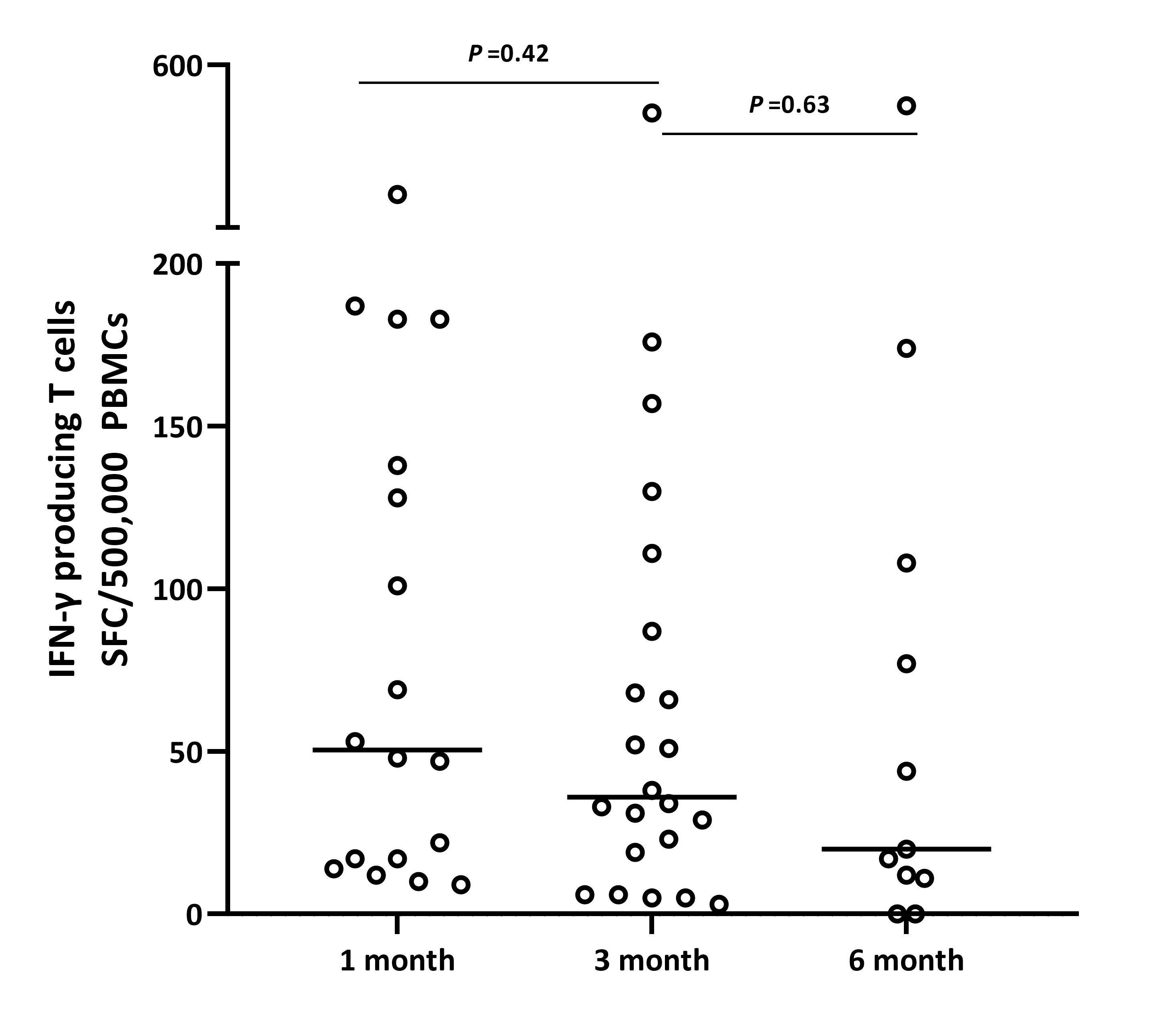

Supplement: Supplementary file 5 — Supporting information [file CTM2-12-e804-s008.tif]

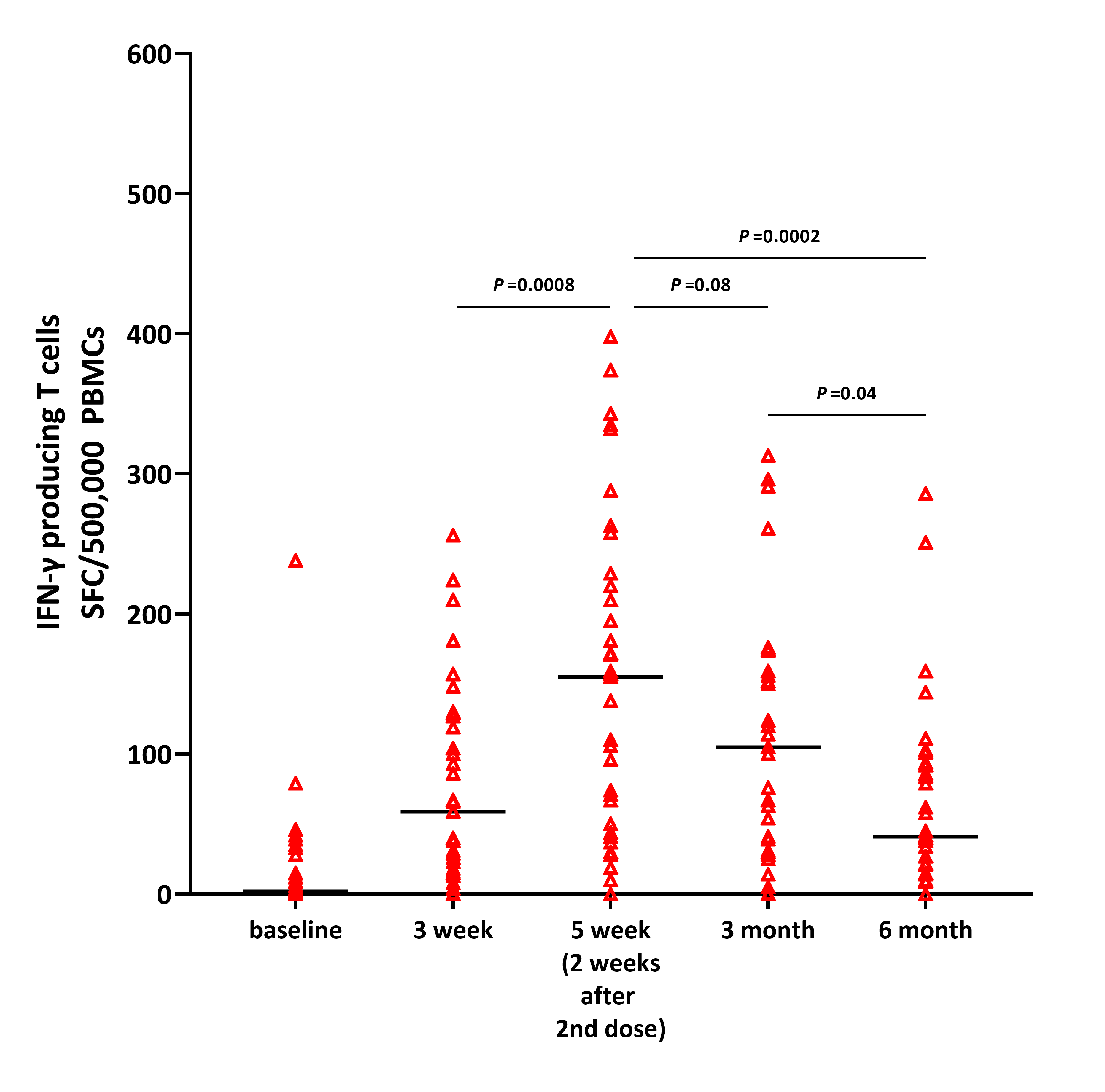

Supplement: Supplementary file 6 — Supporting information [file CTM2-12-e804-s004.tif]

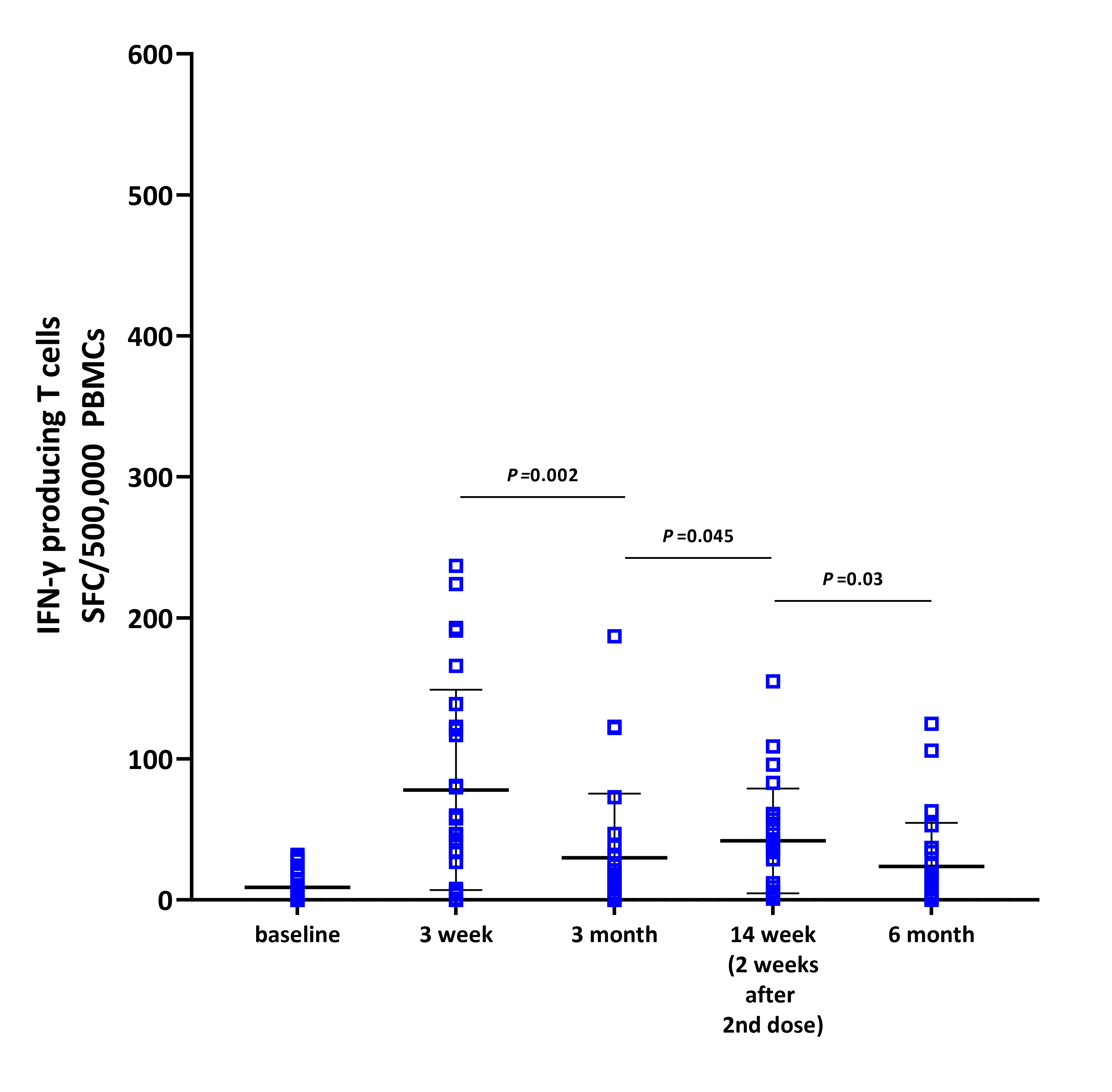

Supplement: Supplementary file 7 — Supporting information [file CTM2-12-e804-s001.tif]

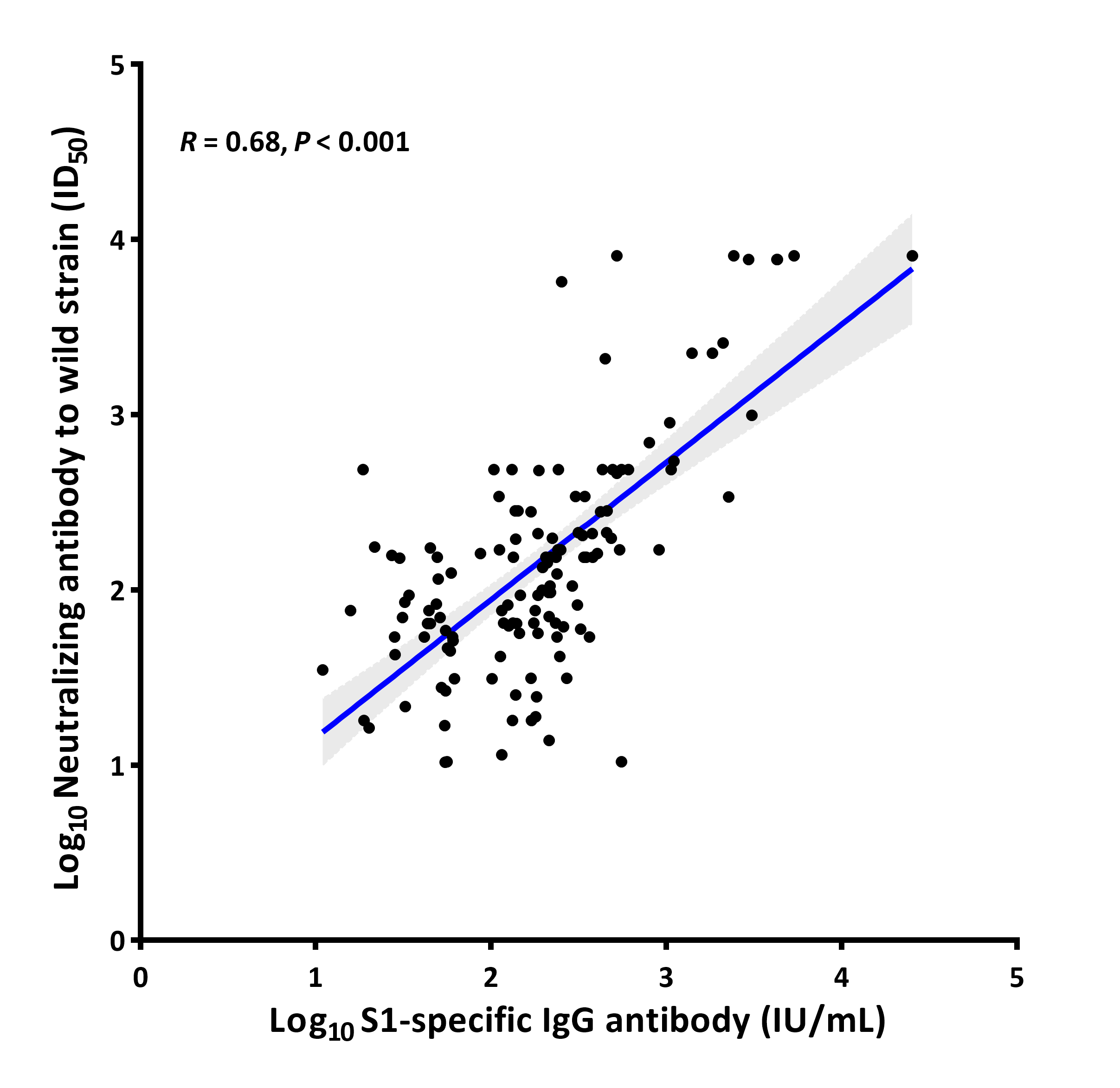

Supplement: Supplementary file 8 — Supporting information [file CTM2-12-e804-s003.tif]

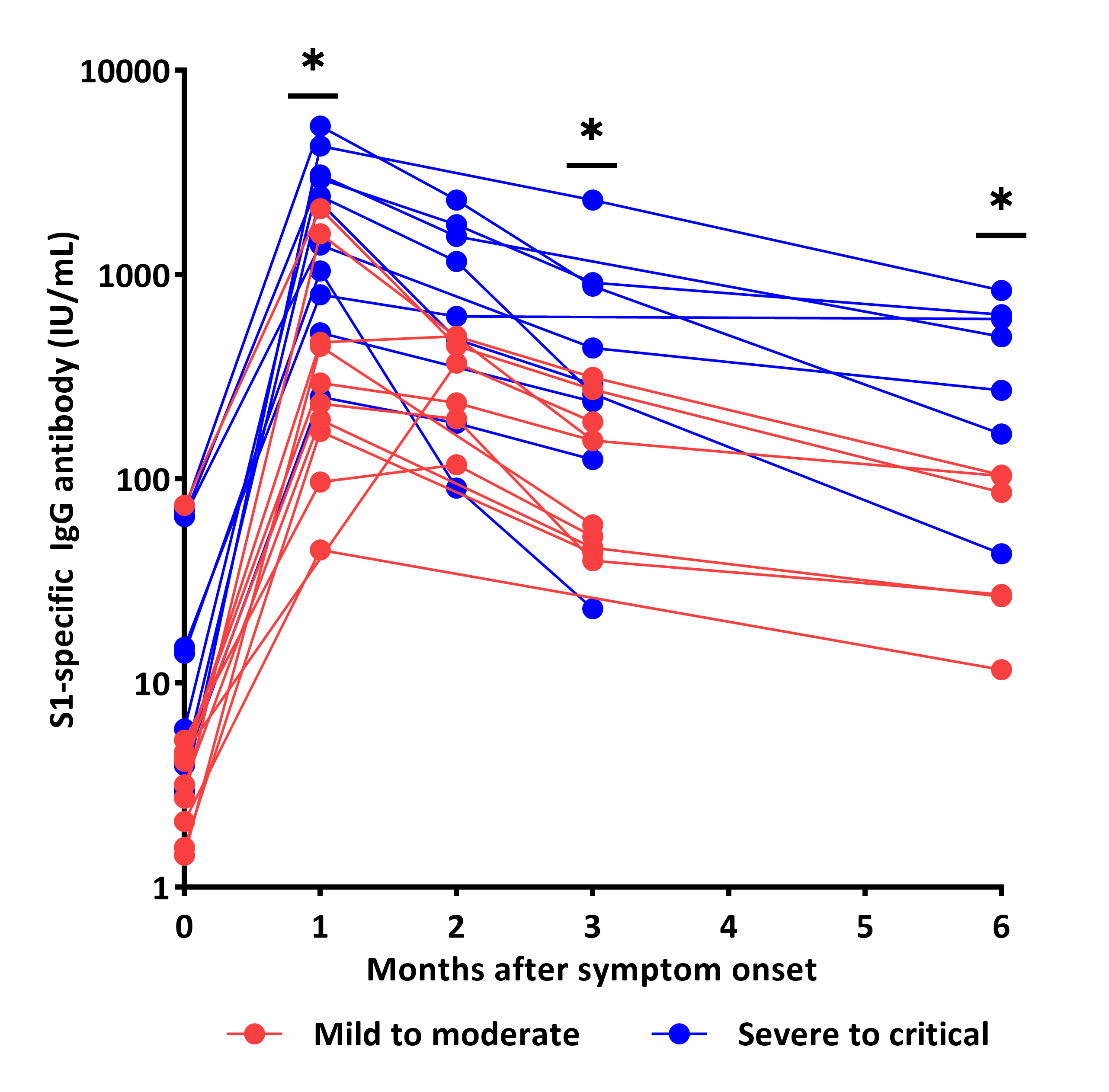

Supplement: Supplementary file 9 — Supporting information [file CTM2-12-e804-s010.tif]

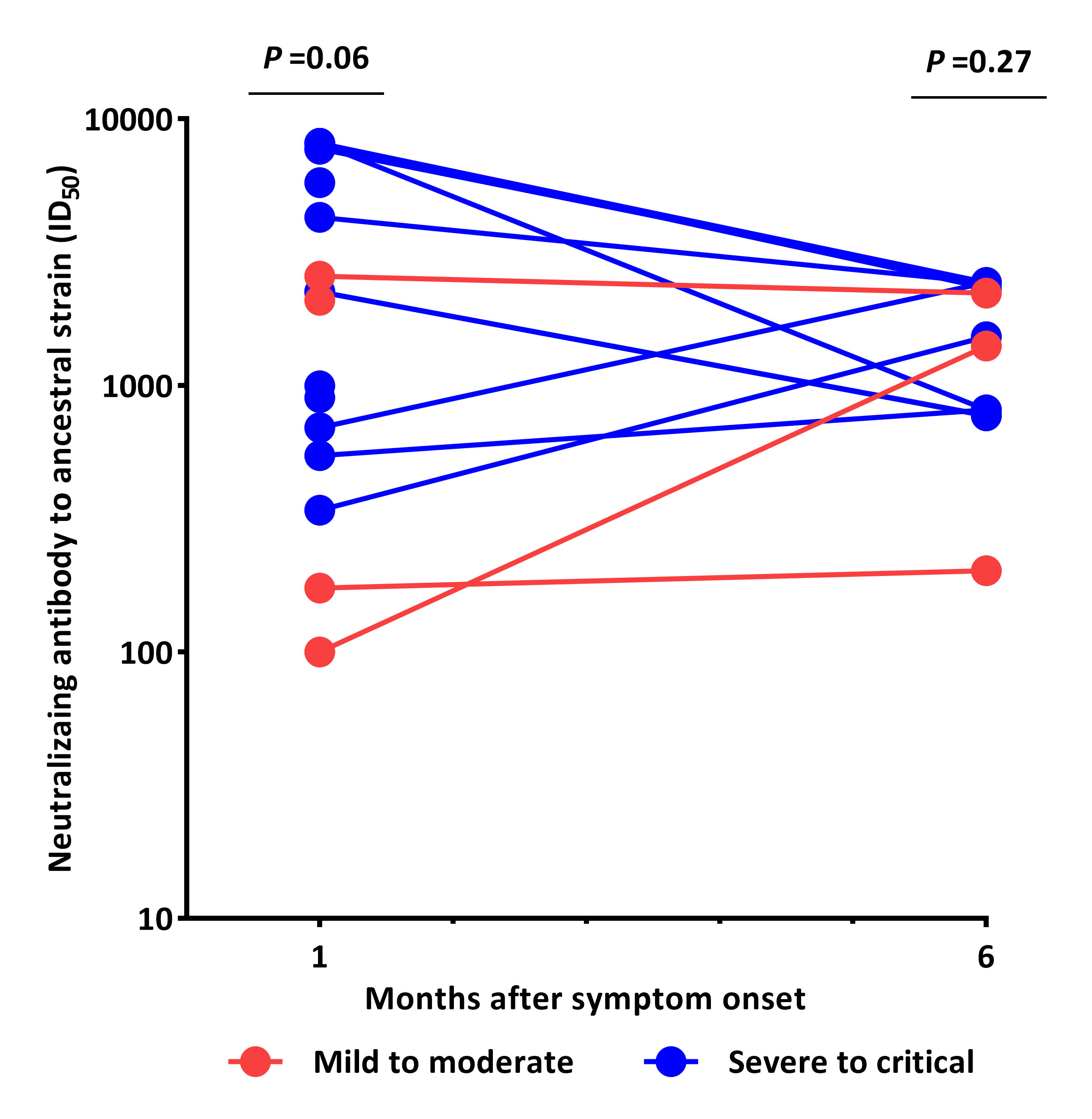

Supplement: Supplementary file 10 — Supporting information [file CTM2-12-e804-s007.tif]

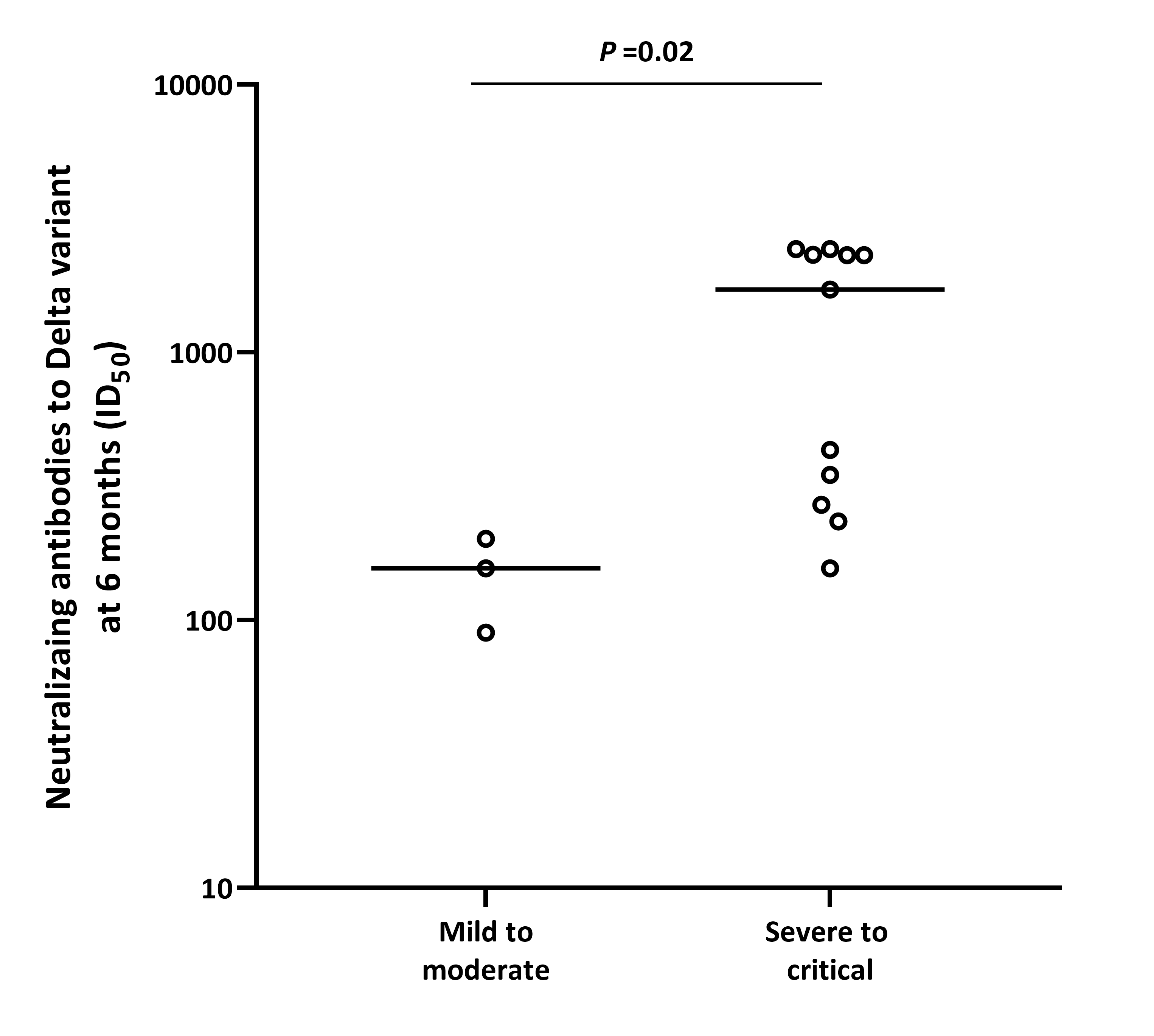

Supplement: Supplementary file 11 — Supporting information [file CTM2-12-e804-s005.tif]
