# Supplementary material for: Breakthrough infections and waning immune responses with ChAdOx1 nCoV‐19 or mRNA vaccine in healthcare workers
Source: Clin Transl Med. 2022 Apr 22;12(4):e804. doi: 10.1002/ctm2.804 (PMC9029012; doi:10.1002/ctm2.804)
Supplement: Supplementary file 12 — Supporting information [file CTM2-12-e804-s011.docx]

**Supplemental Table 1. The infection rate of fully vaccinated HCWs with ChAdOx1 nCoV-19 and mRNA vaccine stratified by days since first vaccination**

| **Time since first vaccination (days)** | **ChAdOx1 nCoV-1** | **mRNA vaccine** | **P value** |
| --- | --- | --- | --- |
| **91-120** | 0.06% (6/9716) | 0.08% (2/2675) | 0.69 |
| **121-150** | 0.01% (1/9710) | 0.09% (2/2289) | 0.10 |
| **151-180** | 0.08% (8/9702) | 0.11% (2/1765) | 0.66 |
| **181-210** | 0.09% (9/9693) | 0.21% (2/965) | 0.26 |
| **211-240** | 0.19% (15/8050) | 0.54% (2/372) | 0.17 |
| **241-300** | 0.63% (50/7950) | 0.55% (2/365) | >0.99 |
| **P for trend** | <0.001 | 0.006 |  |

**Supplementary Figure legends**

**Supplementary Figure 1.** Kinetics of humoral immune responses after SARS-CoV-2 natural infection and COVID-19 vaccination. *A*. Natural infection. *B*. BNT162b2. *C*. ChAdOx1.

**Supplementary Figure 2.** Kinetics of T cell immune responses after SARS-CoV-2 natural infection and COVID-19 vaccination. Horizontal lines indicate the median value. *A*. Natural infection. *B*. BNT162b2. *C*. ChAdOx1.

**Appendix Figure legends**

**Appendix Figure 1.** Correlation between the 50% neutralizing antibody titer and S1-specific IgG antibody titer. Solid line indicates the simple linear regression. Gray shaded area indicates the 95% confidence interval.

**Appendix Figure 2. Antibody responses over 6 months according to the severity of SARS-CoV-2 infection.** **P* < 0.05. *A*. S1-specific IgG antibody. *B*. Neutralizing antibody to ancestral strain. *C*. Neutralizing antibody to delta strain at 6 months.
